# Pediatric Critical Illness Score, Clinical Characteristics and Comprehensive Treatment of Children with Severe Mycoplasma Pneumoniae Pneumonia

**DOI:** 10.3389/fsurg.2022.897550

**Published:** 2022-05-25

**Authors:** Chengchao Fang, Yueyan Mao, Mingfen Jiang, Wei Yin

**Affiliations:** ^1^ Department of Pediatrics, The First People’s Hospital of Linping District, Hangzhou, China; ^2^ Hemodialysis center, The First People’s Hospital of Linping District, Hangzhou, China

**Keywords:** severe Mycoplasma pneumoniae pneumonia, pediatric critical illness score, clinical characteristics, antibiotics, glucocorticoids, comprehensive treatment

## Abstract

**Objective:**

To investigate the clinical characteristics of children with severe Mycoplasma pneumoniae pneumonia (SMPP) and the correlation with pediatric critical illness score (PICS), and to explore the effect of combined treatment with antibiotics and glucocorticoids.

**Methods:**

The medical records of 120 children with SMPP admitted to our hospital from January 2020 to June 2021 were retrospectively analyzed. Children with a PICS score greater than 80 within 24 h of admission were included in the non-critical group, those with a score of 71–80 were included in the critical group, and those with a score of ≤70 were included in the extremely critical group. The relevant clinical data and examination indicators of the three groups of children were intercepted and compared. Univariate and multifactorial logistic regression analyses were performed to analyze the correlation between clinical characteristics of children with SMPP and PICS. According to the different treatment methods, the children were subdivided into the control group (*n* = 54) who received antibiotics alone and the comprehensive group (*n* = 66) who received antibiotics combined with glucocorticoid therapy. The erythrocyte sedimentation rate (ESR), inflammation and immune indexes, symptom relief or disappearance time, hospitalization days, and clinical efficacy were compared between the two groups before and after treatment.

**Result:**

Within 24 h of admission, among the 120 children with SMPP, 79 had PICS >80, 32 had PICS 71–80, and 9 had PICS ≤70. Before discharge, among the 120 children with SMPP, 99 had PICS >80, 17 had PICS 71–80, and 4 had PICS ≤70. Univariate analysis showed that there were no significant differences in gender ratio, ratio of fever duration >10 days, age and WBC among the three groups (*p *> 0.05), the differences in the ratio of abnormal ECG, the ratio of ≥2 pathogenic infections, the ratio of ≥2 systemic damages, CRP levels, and D-dimer levels were statistically significant when compared among the three groups (*p *< 0.05). Multivariate Logistic regression analysis showed that the number of Co-systemic damages and the level of D-dimer were negatively correlated with PICS classification (*p *< 0.05). After medication, ESR, CRP, IL-6, and CD8+ levels decreased and CD4+ and CD4+/CD8+ levels increased in both the control and comprehensive groups, and all changes were significant in the comprehensive group compared with the control group (*p *< 0.05). The antipyretic time, cough relief time, disappearance time of lung rales and hospitalization days in the comprehensive group were shorter than those in the control group (*p *< 0.05). The total effective rate of the comprehensive group (95.45%) was better than that of the control group (83.33%) (*p *< 0.05).

**Conclusion:**

PICS can effectively reflect the clinical characteristics of children with SMPP. The comprehensive treatment effect of azithromycin combined with glucocorticoid is significantly better than that of azithromycin alone. It can effectively reduce the level of inflammation in children with SMPP, improve the immune function of children, and accelerate clinical recovery. It has promotion value.

## Introduction

Mycoplasma pneumoniae pneumonia (MPP) has gradually become one of the most common manifestations of community-acquired pneumonia in children. It refers to respiratory disease caused by Mycoplasma pneumoniae (MP) infection in children that are mostly mild and self-limiting, or respond well to macrolide antibiotics (1, 2). However, in recent years, pathogen resistance due to long-term antimicrobial drug therapy has increased, and the number of cases of severe Mycoplasma pneumoniae pneumonia (SMPP) has been increasing, with children having special clinical manifestations, obvious immune disorders, and poor efficacy of macrolide antibiotic therapy alone. Severe cases can cause necrotizing pneumonia (3), hepatitis (4), encephalitis (5), myocarditis (6), hemophagocytic syndrome (7), and even life-threatening in children. Early detection of severe cases and appropriate treatment can prevent or reduce the occurrence of serious sequelae or death.

Pediatric critical illness score (PICS) selects 10 indicators to score critical cases in children, all of which use quantitative data or objective indicators obtained from laboratory examination or physical examination, and the first score can accurately reflect the severity of children’s illness, has a certain judgment value for the prognosis of the disease (8).

At present, there are few reports on the correlation between clinical features of SMPP and PICS, and there is also a lack of objective indicators and reports that can alert disease progression. In this study, 120 children with SMPP were included and grouped by PICS, and the relevant clinical data and examination indicators of different groups were retrospectively analyzed, in order to systematically evaluate the clinical characteristics of children with SMPP and their correlation with PICS scores, and to further clarify the guiding significance of PICS for the diagnosis and treatment of SMPP. In addition, some scholars have reported that the combination of drugs has a significant effect. This study conducted a secondary group analysis. According to the different treatment methods, the children were divided into the control group who received antibiotics alone and the comprehensive group who received antibiotics combined with glucocorticoid therapy. The erythrocyte sedimentation rate (ESR), inflammatory and immune indexes, symptom relief or disappearance time, hospitalization days, and clinical efficacy were analyzed and compared between the two groups before and after medication. The summary and analysis are as follows.

## Materials and Methods

### Research Object

This study was a retrospective study, and the subjects were the medical records of 120 children with SMPP admitted to our hospital from January 2020 to June 2021. Inclusion criteria: ≤12 years old. Complete medical records. The symptoms and related examinations of the children met the diagnostic criteria for MPP in the “Handbook of Diagnosis and Treatment of Common Respiratory Diseases in Children”, and on this basis met the following points: ① With symptoms of dyspnea and tachycardia; ② Accompanying hypoxemia; ③ Fever (armpit temperature) >38.5°C for more than 1 week; ④ Complications such as pleural effusion and atelectasis occured, and imaging data showed that more than two-thirds of the thoracic segments were involved; ⑤ Incorporate at least one system damage. With antibiotics or combined glucocorticoids in hospital. All children were evaluated by PICS within 24 h of admission. Exclusion criteria: Children with congenital developmental defects or immunodeficiency. Children with severe blood disease or cardiovascular disease. Children who had undergone tracheotomy, tracheal intubation, and mechanically assisted ventilation before admission. Severely malnourished children.

### Research Method

Through literature search, we collected and sorted out the clinical characteristics that may affect PICS in children with SMPP. Including gender, abnormal ECG, whether or not with ≥2 pathogenic infections, whether with ≥2 kinds of systemic damages, duration of fever, age, C-reactive protein (CRP), white blood cell count (WBC), plasma D-dimer level. Univariate analysis and multivariate Logistic regression analysis were performed to analyze the correlation between the above indicators and PICS in children with SMPP.

All the children were divided into the control group (*n* = 54) who received antibiotics alone and the comprehensive group (*n* = 66) who received antibiotics combined with glucocorticoids according to the different treatment methods. Among them, the children in the control group received azithromycin (Shenyang No. 1 Pharmaceutical Factory of Northeast Pharmaceutical Group, H20000426) 10 mg·kg^−1^·d^−1^ intravenous drip for 7 days on the basis of routine phlegm-relieving, cough-relieving and symptomatic and supportive treatment. The comprehensive group received methylprednisolone sodium succinate (Pfizer Pharmaceutical Co., Ltd., H31020310) 10 mg·kg^−1^·d^−1^ intravenous drip for 5 days on the basis of the control group. The improvement of ESR, inflammation and immune indexes, time of symptom relief or disappearance, length of hospitalization, and clinical efficacy of the two groups were collected and sorted. Through analysis and comparison, the comprehensive therapeutic effect of antibiotics and glucocorticoids was discussed.

### Research Indicators

PICS grouping: PICS of children within 24 h of admission and before discharge were collected and collated, including heart rate, pH, blood sodium, blood potassium, blood pressure, hemoglobin, respiratory rate, urea nitrogen, creatinine, and oxygen saturation 10 items, each with 10 points, totaling 100 points (9). Children with a PICS score >80 within 24 h of admission were included in the non-critical group, those with a score of 71–80 were included in the critical group, and those with a score ≤70 were included in the extremely critical group. Criteria for determining abnormal ECG: The ECG examination within 72 h of admission was collected and collated, and if the ECG indicated atrial fibrillation, ventricular fibrillation, paroxysmal tachycardia and other arrhythmic signs, the ECG was determined to be abnormal.

Criteria for determining pathogenic infection and systemic damage: Blood culture of pathogenic bacteria (including adenovirus, influenza A virus, Mycoplasma pneumoniae, herpes simplex virus, IgM antibody, Chlamydia pneumoniae, etc.), sputum culture and drug sensitivity test results within 24 h of admission were collected and collated, and if the cumulative number of viral or bacterial infections was ≥2, the child was judged to have a combination of ≥2 pathogenic infections. The types of systemic damage (including electrolyte disorders, liver function damage, gastrointestinal bleeding, myocarditis, central nervous system infection, etc.) that appeared within 72 h of admission were collected and collated, and if the cumulative systemic damage was ≥2, the child was judged to have a combination of ≥2 systemic damages.

Serological indexes: The results of venous blood sampling before drug administration were collected and collated, including CRP (rate scattering turbidimetric method, determined by Mérieux VIDAS automatic immunoassay analyzer), WBC (blood cell count method, determined by Mindray BC-5120 automatic blood cell analyzer), plasma D-dimer (immunoturbidimetric method, determined by Hysenmecom CA-1500 automatic coagulometer, Japan), ESR (measured by PUC-2068A hematocrit meter, purchased from Beijing Pulang New Technology Co., Ltd.), interleukin-6 (IL-6) (immunofluorescence method, kit purchased from Guangzhou Baochuang Biotechnology Co., Ltd.), immunological indicators: CD4^+^, CD8^+^, CD4^+^/CD8^+^ (measured by BD FACSCalibur multicolor flow cytometer, mouse anti-human CD4-R D1, CD8-ECD monoclonal antibodies were purchased from Beckman). The venous blood results of the children after 14 days of medication were collected and sorted, including ESR, CRP, IL-6, CD4^+^, CD8^+^, CD4^+^/CD8^+^.

Symptoms resolution: The fever reduction time, cough relief time, pulmonary rales disappearance time, and hospitalization days of children receiving different treatment plans were collected and sorted.

Clinical efficacy: The clinical efficacy of the children after 14 days of medication were compared, The judgment criteria were evaluated with reference to the “Guidelines for Clinical Research on Antibacterial Drugs”. Healed: Symptoms, signs, sputum culture, chest X-ray, WBC and other indicators have returned to normal; Markedly effective: symptoms and signs basically disappeared, sputum culture returned to negative, X-ray showed that the inflammation was basically absorbed, and WBC returned to normal, etc.; Effective: symptoms and signs improved significantly, and WBC returned to normal; ineffective: no obvious improvement or aggravation of the condition.

### Statistical Method

The analysis software was SPSS22.0. All relevant data were in accordance with normal distribution by K-S test. The enumeration data were expressed as percentage (%), and the continuous correction *χ*^2^ test was performed. The normally distributed measurement data were expressed as mean ± standard deviation (*M* ± *SD*), the *t*-test was used for comparison between groups. Variables with statistically significant differences between multiple groups in univariate analysis required further multivariate logistic regression analysis. Statistical difference was expressed as *p *< 0.05.

## RESULT

### PICS Classification of 120 Children with SMPP

**Figure 1:**
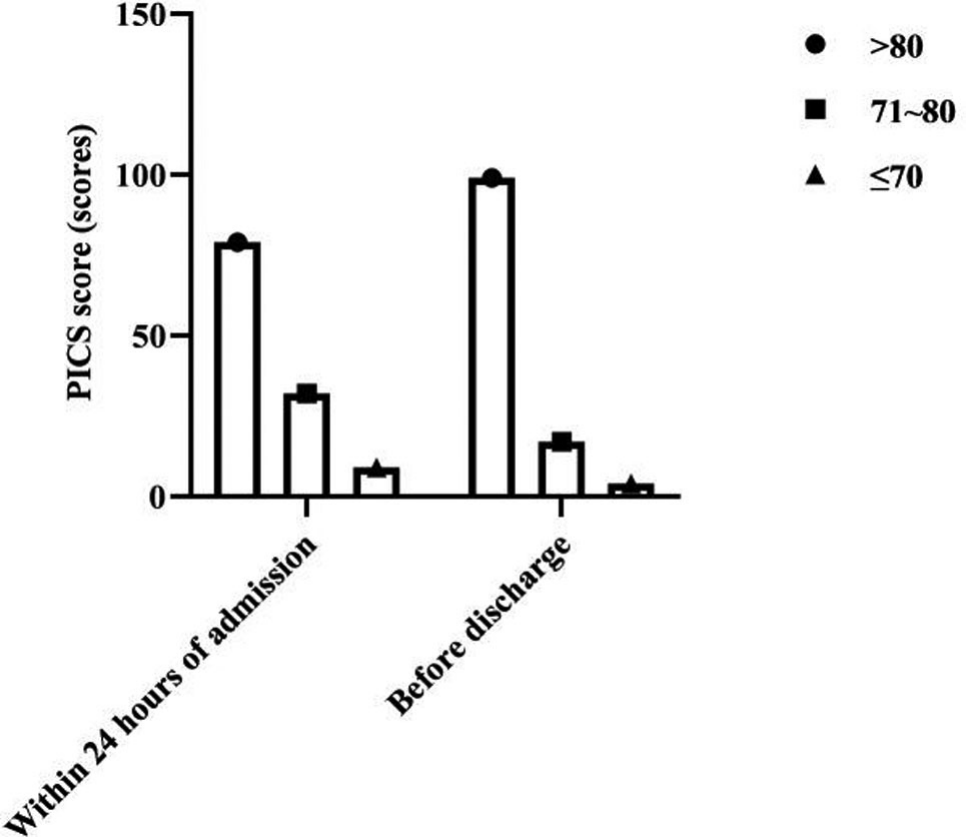
PICS classification of 120 children with SMPP.

Within 24 h of admission, among the 120 children with SMPP, 79 had PICS >80, 32 had PICS 71–80, and 9 had PICS ≤70. Before discharge, among the 120 children with SMPP, 99 had PICS >80, 17 had PICS 71–80, and 4 had PICS ≤70. As seen in Figure 1.

### Analysis of Clinical Characteristics of 120 Children with SMPP

Univariate analysis showed that there were no significant differences in gender ratio, ratio of fever duration >10 days, age and WBC among the three groups (*p *> 0.05), the differences in the ratio of abnormal ECG, the ratio of ≥2 pathogenic infections, the ratio of ≥2 systemic damages, CRP levels, and D-dimer levels were statistically significant when compared among the three groups. As seen in Table 1.

**Table 1:** Analysis of clinical characteristics of 120 children with SMPP.

Indicator	Non-critical group (*n* = 79)	Critical group (*n* = 32)	Extremely critical group (*n* = 9)	*χ*^2^/*F*	*p*
Male /Female	43/36	19/13	5/4	0.226	0.893
Abnormal ECG (*n*, %)	11 (13.92)	11 (34.38)	6 (66.67)	15.538	<0.001
≥2 pathogenic infections (*n*, %)	10 (12.64)	12 (37.50)	6 (66.67)	18.070	<0.001
≥2 systemic damages (*n*, %)	29 (36.71)	25 (78.13)	9 (100.00)	24.468	<0.001
Fever duration >10 days (*n*, %)	7 (8.86)	4 (12.50)	3 (33.33)	4.725	0.094
Age (years old)	5.63 ± 1.47	5.63 ± 1.34	5.67 ± 1.41	0.003	0.997
CRP (mg/L)	45.14 ± 9.12	60.01 ± 14.21	72.56 ± 18.56	35.810	<0.001
WBC (×10^9^/L)	14.24 ± 3.32	15.92 ± 4.48	15.65 ± 4.50	2.541	0.083
D-dimer (mg/L)	3.21 ± 0.86	8.46 ± 0.90	12.41 ± 1.20	694.400	<0.001

### Multivariate Logistic Regression Analysis of PICS Classification and Clinical Characteristics in 120 Children

Multivariate Logistic regression analysis showed that the number of Co-systemic damages and the level of D-dimer were negatively correlated with PICS classification (*p *< 0.05). As seen in Table 2.

**Table 2:** Multivariate Logistic regression analysis of PICS classification and clinical characteristics in 120 children.

Indicator	*r*	*p*	Assignment	PICS classification
ECG examination	0.520	0.187	1 = normal, 2 = sometimes abnormal, 3 = abnormal	Grade A: PICS > 80, non-critical; Grade B: PICS 71–80, –critical, Grade C: PICS ≤70, extremely critical
Co-pathogenic infections	−0.80	0.101	1 = 1 type, 2 = 2 types, 3 = 3 types and more	
Co-systemic damages	−1.53	0.023	1 = none, 2 = 1 type, 3 = 2 types, 4 = 3 types and more	
CRP	−0.90	0.085	1 ≤ 8. 20 mg/L, 2 = 8. 20–40. 00 mg/L, 3 ≥ 40. 0 mg/L	
D-Dimer	−1.44	0.030	1 ≤ 0. 20 mg/L, 2 = 0. 20–2. 00 mg/L, 3 ≥ 2. 00 mg/L	

### Analysis of ESR, CRP and IL-6 Levels in 120 Children

**Figure 2:**
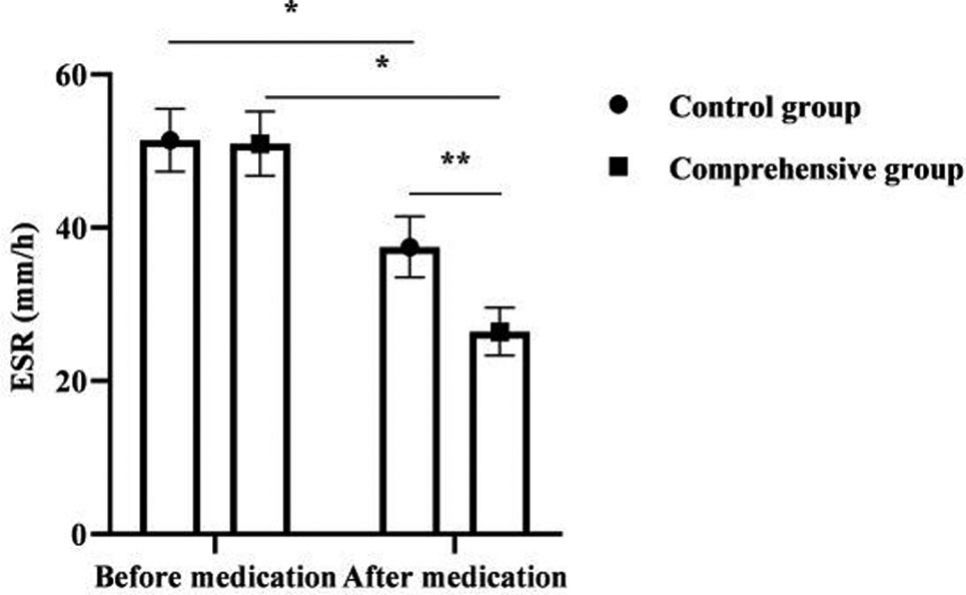
Analysis of ESR, CRP and IL-6 levels in 120 children. Note: * was *p *< 0.05 for the comparison before and after medication; ** was *p* < 0.05 for the comparison after medication.

After medication, ESR, CRP, and IL-6 levels decreased in both groups, with significant changes in the comprehensive group compared to the control group (*p *< 0.05). As seen in Figure 2.

### Analysis of Immune Indexes in 120 Children

**Figure 3:**
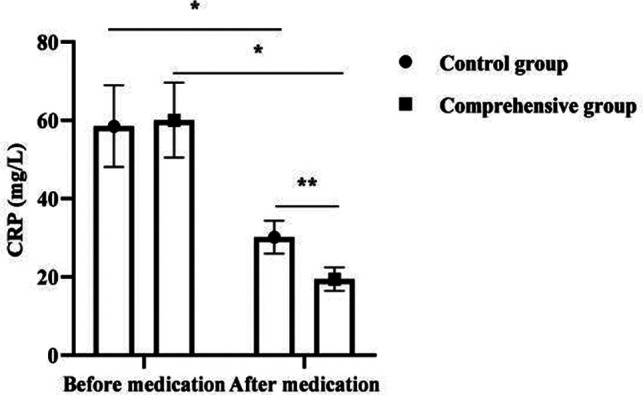
Analysis of immune indexes in 120 children. Note: * was *p *< 0.05 for the comparison before and after medication; ** was *p *< 0.05 for the comparison after medication.

After medication, CD4+ and CD4+/CD8+ levels increased and CD8+ levels decreased in both groups, with significant changes in the comprehensive group compared to the control group (*p *< 0.05). As seen in Figure 3.

### Analysis of Symptom Resolution in 120 Children

**Figure 4:**
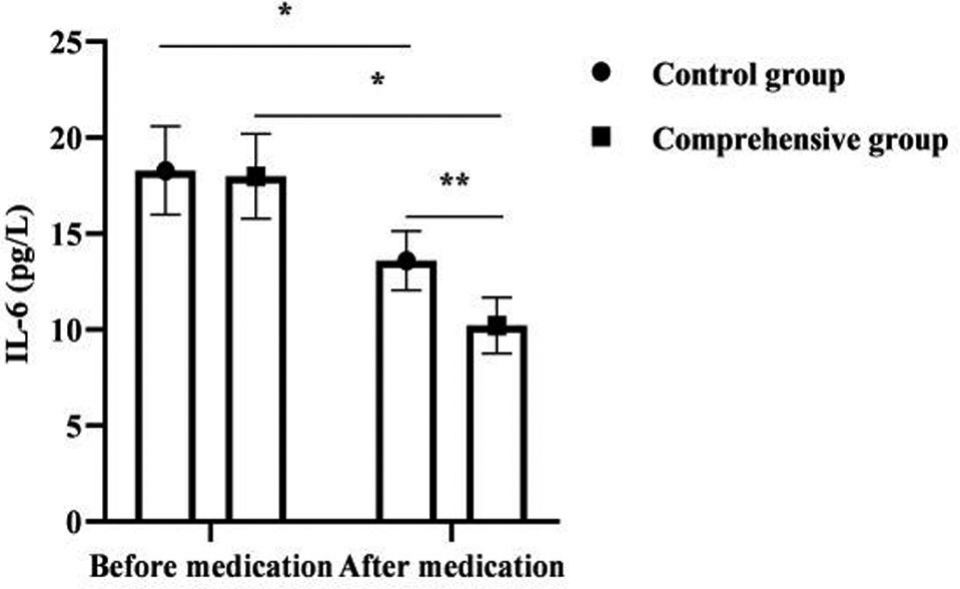
Analysis of symptom resolution in 120 children. Note: * was *p *< 0.05 for comparison between groups.

The antipyretic time, cough relief time, disappearance time of lung rales and hospitalization days in the comprehensive group were shorter than those in the control group (*p *< 0.05). As seen in Figure 4.

### Clinical Efficacy Analysis of 120 Children

The total effective rate of the comprehensive group (95.45%) was better than that of the control group (83.33%) (*p *< 0.05). As seen in Table 3.

**Table 3:** Clinical efficacy analysis of 120 children.

Group	Healed	Markedly effective	Effective	Ineffective	Total effective
Control group (*n* = 54)	30 (55.56)	8 (14.81)	7 (12.96)	9 (16.67)	45 (83.33)
Comprehensive group (*n* = 66)	44 (66.66)	14 (21.21)	5 (7.58)	3 (4.55)	63 (95.45)
*χ* ^2^	1.551	0.812	0.958	4.849	4.849
*p*	0.213	0.368	0.328	0.028	0.028

## Discussion

Children are susceptible to MPP, and their clinical manifestations vary. If the disease is not effectively controlled in a timely manner, the disease may continue to progress to SMPP or may involve multiple organs and systems throughout the body, seriously threatening their life safety (10). As such, how to quickly and effectively assess the condition of MPP and promptly diagnose and treat it has become a hot issue in pediatric clinics. PCIS can effectively reflect the severity of the child’s condition through a comprehensive evaluation of 10 indicators, such as heart rate and pH etc. Wang et al. (11), a domestic scholar, used PCIS to evaluate the criticality of severe pneumonia in infants and children and found that PCIS could accurately determine the progression of the child’s disease, and the lower the score, the greater the proportion of multi-organ damage occurred, and by dynamically observing the PCIS score, there was a significant correlation between the improvement of the patient’s condition and the PCIS score. In addition, some authors (12) found that the PCIS score can be used to assess the condition of children with hand, foot and mouth disease complicated with encephalitis, in which heart rate, blood pressure and renal function can effectively predict the risk of death with a high specificity and sensitivity. However, with the widespread development of clinical applications, the lack of coagulation indicators and poor sensitivity to the severity of the disease have become increasingly prominent in PICS, making it questionable whether the PICS score can comprehensively assess the severity of SMPP.

This study investigated the clinical characteristics of children with SMPP and their correlation with PICS. By comparing the clinical characteristics of children with different PICS score groups, it was found that the number of combined systemic damage and the level of D-dimer were negatively correlated with PICS classification. The incidence of MPP infection in children with other pathogens is as high as 50%, and it can enhance the virulence of bacteria and viruses, resulting in SMPP (13). Song et al (14) found that the proportion of mixed infection in children with SMPP in Beijing was significantly higher than that in children without SMPP. Among them, 25% were infected with two pathogens; the occurrence of mixed infections often leads to prolonged disease course, increased drug use, and greatly increases the risk of developing multi-system and multi-organ damage. This study suggests that the number of combined systemic damage is negatively correlated with the PICS classification, which needs to be paid attention to in clinical practice. With the aggravation of MPP, the thrombin and fibrin in the children’s body are continuously activated, leading to an imbalance between the coagulation and anticoagulation systems, resulting in a hypercoagulable state, which further leads to clinical symptoms and aggravates the damage to other system functions (15). D-dimer is an important indicator reflecting coagulation function. It is a sensitive marker of thrombus formation in the vascular circulatory system and can reflect the activity of thrombin and plasmin (16). This study found that the level of D-dimer was negatively correlated with the PICS classification, indicating that children with SMPP have different degrees of blood hypercoagulation, and the PCIS score can effectively reflect the coagulation function of children with SMPP and thus determine the progress of the disease. For example, one study (17) reported that Mycoplasma pneumoniae infection causes vascular embolism in children and that a hypercoagulable state of the blood is its main cause. This is important to guide the anticoagulation treatment and prognostic assessment of the child. Inflammatory mediators play an important role in the pathogenetic progression of MPP. Among them, CRP, an acute phase-responsive protein, can be extremely increased in case of infection or inflammation and is associated with criticality of the disease (18). However, the present investigation showed no correlation between PCIS scores and CRP levels, which may be related to the younger age of the children included in the study and the weaker CRP synthesis ability.

It is reported that the comprehensive treatment of antibiotics and glucocorticoids can significantly improve the clinical effect of MPP in children (19). Glucocorticoids are commonly used clinical immunomodulators, which can play a variety of pharmacological effects such as immunomodulation, anti-allergy, and anti-inflammatory. The present results showed that the combined group treated with combined glucocorticoids had significantly lower ESR, CRP, IL-6, and CD8^+^ levels and significantly higher CD4^+^ and CD4^+^ /CD8^+^ levels than the control group after medication. As a typical marker of disease activity and inflammatory infection, ESR can directly reflect the dynamic changes and development of the disease (20), and the ESR of SMPP children is significantly higher than that of ordinary MPP children. In the present results, after treatment, the ESR of the comprehensive group was significantly lower than that of the control group, indicating that the combined treatment of the two was beneficial to disease control. It was previously believed that the pathogenesis of SMPP is mainly related to the direct action of pathogens and the indirect action of pathogens stimulating inflammatory immune responses (21). When the organism is infected with pneumonia, mycoplasma enters the lung through the airway and stimulates macrophages at the alveoli, producing inflammatory cytokines such as CRP and IL-6, and then stimulates various cytokines in the lungs and induces inflammatory cells in the peripheral circulation to enter the alveoli and interstitium, which in turn produce a large number of cytokines and inflammatory mediators, further aggravating the inflammatory response of the organism (22). Mei Yuxia (23) found that azithromycin combined with glucocorticoids for SMPP resulted in a significant reduction in inflammatory factor levels, and the present results also showed this effect. In addition, mycoplasma is capable of causing disruption of the normal ratio of T lymphocyte subsets and inducing reduced and disturbed cytokine production (24). It is common that the dynamic balance between CD4+ and CD8+ is disrupted, leading to pathological immune damage. The improvement of immune function was more significant in the comprehensive group after medication in this result, suggesting that the combination of both treatments is more helpful in the correction of the organism’s immune dysfunction. The results of this study also showed that the time to fever resolution, cough relief, disappearance of pulmonary rales and days of hospitalization were shorter in the comprehensive group than in the control group. The total effective rate of the comprehensive group was better than that of the control group. It indicates that the combination of antibiotics and glucocorticoids can promote the improvement of clinical manifestations in children with SMPP, thus reducing their suffering and increasing their tolerance.

## Conclusion

PICS can effectively reflect the clinical characteristics of children with SMPP. The comprehensive treatment effect of azithromycin combined with glucocorticoid is significantly better than that of azithromycin alone. It can effectively reduce the level of inflammation in children with SMPP, improve the immune function of children, and accelerate clinical recovery. It has promotion value.

## Data Availability

The original contributions presented in the study are included in the article/Supplementary Material, further inquiries can be directed to the corresponding author/.
